# Quantifying dispersal between two colonies of northern elephant seals across 17 birth cohorts

**DOI:** 10.1371/journal.pone.0288921

**Published:** 2023-11-30

**Authors:** Richard Condit, Brian Hatfield, Patricia A. Morris, Daniel P. Costa

**Affiliations:** 1 Department of Ecology and Evolutionary Biology, University of California, Santa Cruz, Santa Cruz, CA, United States of America; 2 Independent Researcher, United States of America; 3 Institute for Marine Sciences, University of California, Santa Cruz, Santa Cruz, CA, United States of America; Surf Life Saving Australia, AUSTRALIA

## Abstract

Dispersal drives extinction-recolonization dynamics of metapopulations and is necessary for endangered species to recolonize former ranges. Yet few studies quantify dispersal and even fewer examine consistency of dispersal over many years. The northern elephant seal (*Mirounga angustirostris*) provides an example of the importance of dispersal. It quickly recolonized its full range after near extirpation by 19^th^ century hunting, and though dispersal was observed it was not quantified. Here we enumerate lifetime dispersal events among females marked as pups at two colonies during 1994-2010, then correct for detection biases to estimate bidirectional dispersal rates. An average of 16% of females born at the Piedras Blancas colony dispersed northward 200 km to breed at Año Nuevo, while 8.0% of those born at Año Nuevo dispersed southward to Piedras Blancas. The northward rate fluctuated considerably but was higher than southward in 15 of 17 cohorts. The population at Piedras Blancas expanded 15-fold during the study, while Año Nuevo’s declined slightly, but the expectation that seals would emigrate away from high density colonies was not supported. During the 1990s, dispersal was higher away from the small colony toward the large. Moreover, cohorts born later at Piedras Blancas, when the colony had grown, dispersed no more than early cohorts. Consistently high natal dispersal in northern elephant seals means the population must be considered a single large unit in terms of response to environmental change. High dispersal was fortuitous to the past recovery of the species, and continued dispersal means elephant seals will likely expand their range further.

## Introduction

Dispersal and immigration are vital statistics of populations. Besides affecting gene flow and evolution, dispersal regulates metapopulation dynamics, and by overcoming local extinctions it can be crucial in species recovery from population crashes [1–3]. The northern elephant seal offers a clear example. Following near extermination by 19^th^-century hunters, it readily dispersed to reoccupy its former range [4–6]. During 50 years of research in California, we observed dispersal and documented new colonies formed by immigrants [7], but quantifying dispersal has been difficult, as is often the case in large animals that move long distances [8, 9]. Without precise estimates of rates of movement, we do not know whether dispersal continues now that the range is reestablished, and we cannot compare the importance of dispersal in elephant seals to other species [10, 11]. Here we fill the gap by quantifying dispersal rate in two directions in 17 consecutive birth cohorts. In a review of emigration rates, only four studies included more than 17 years [12].

Seals and other colonial animals have an important advantage in measurements of dispersal because breeding locations are discrete, so dispersal is a binomial process, either happening or not, and there are limited locations that must be searched to identify migrants. These features contrast with many terrestrial birds that have been the subject of dispersal research, where every individual disperses some distance [1, 9, 13]. We have been applying lifetime marks to female elephant seals for 30 years at two major colonies in central California, Año Nuevo and Piedras Blancas, and recording sightings of those animals at both colonies every year, so we can now be precise about dispersal in both directions. What percent of females born at one colony moved to the other to breed? Is there asymmetry in direction, with females more likely to move northward or southward? Our observations include the early phase of expansion at the Piedras Blancas colony, and so we can ask whether emigration increased as the colony expanded. Meantime, since Año Nuevo was large throughout, we can ask whether dispersal was greater from the large colony to the small, addressing the hypothesis that colony size drives emigration [12].

## Materials and methods

### Ethics statement

Seal observations were authorized under Marine Mammal Research Permits 347, 404, 486–1506, 486–1790, 684, 704, 705, 774–1437, 774–1714, 836, and 14097; National Marine Sanctuary Permits GFNMS/MBNMS/ CINMS-04–98, MULTI-2002–003, MULTI-2003- 003, and MULTI-2008–003; and Marine Mammal Protection Act Permit 486. Access to park land was granted by the California Department of Parks and Recreation.

### Study sites and colonies

**Fig 1:**
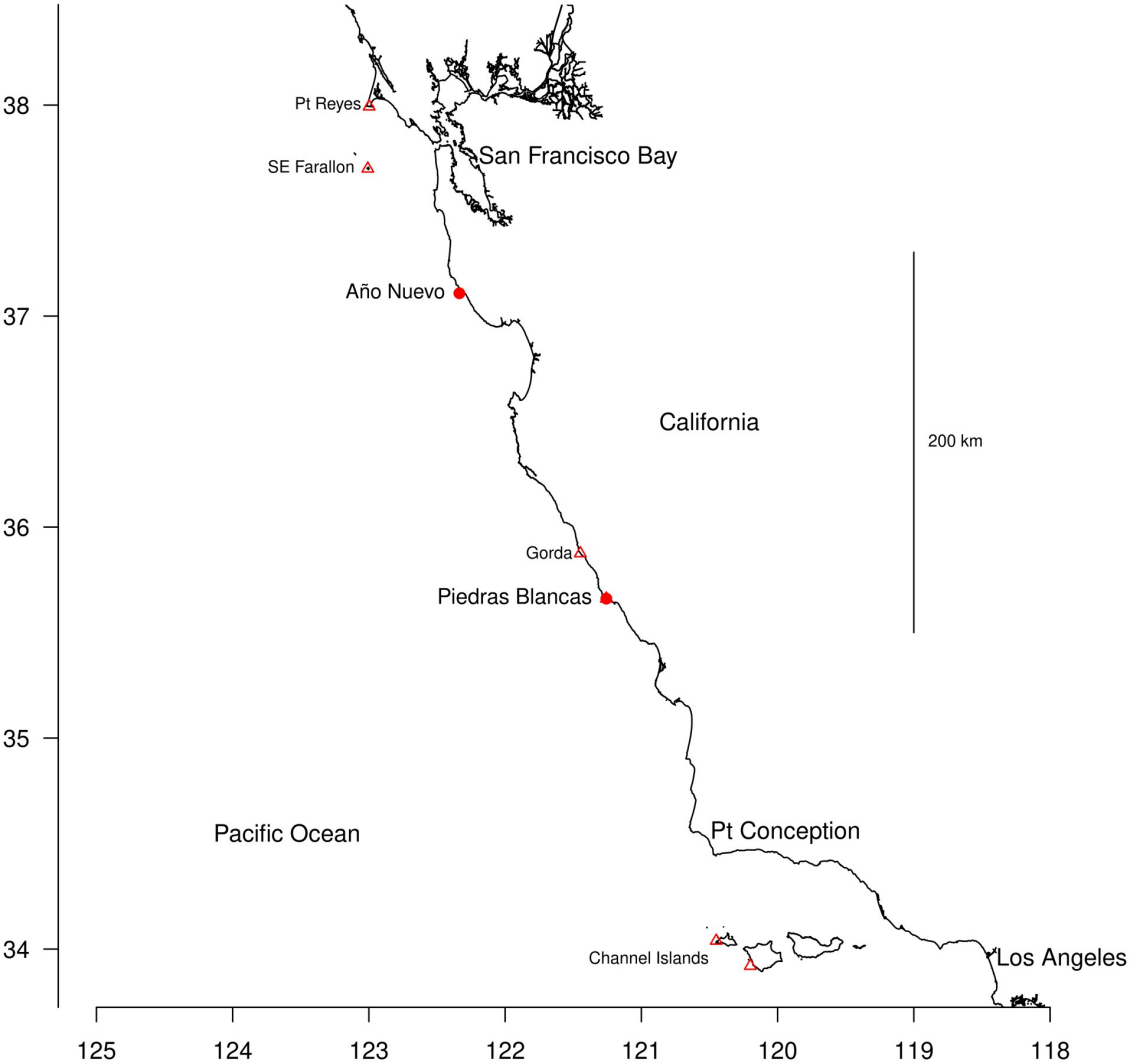
Map showing colony locations. The two study colonies at Año Nuevo and Piedras Blancas are marked with filled red circles. Other nearby colonies are marked with small triangles. The closest colonies at Southeast Farallon and Gorda are small, with only ∼ 100 breeding females each; Point Reyes has ∼900; the Channel Islands are enormous, with > 20, 000 at San Miguel and Santa Rosa combined [6]. There are three more large colonies much further south and another small one further north [6]. Axes show degrees latitude and longitude.

Our study sites at Point Piedras Blancas and Año Nuevo (35.7°, 37.1° N in California) are the largest northern elephant seal colonies on the mainland, providing accessibility and large samples. Other large colonies are on islands and difficult to access (Fig 1). At both sites, female elephant seals gather in large groups on flat sand beaches every winter and give birth to a single pup. Pups are weaned an average of 26 days after birth when mothers depart to forage [14], and weaned pups are easily approached and tagged on the beach before they go to sea [15]. The Año Nuevo colony has had pups every winter since 1961. It expanded rapidly until 1995, then from 1995–2010, annual pup production declined slowly from 2700 to 2100 [6]. The Piedras Blancas colony first had pups in 1992 then grew from 300 pups in 1994 to 4400 in 2010 [6].

### Tagging and lifetime breeding records

Plastic sheep tags were inserted in the interdigital webbing of the hind flippers of weaned pups [15]; since 1998, most tags deployed were Jumbo Roto tags. On average, 21% of weaned pups were tagged at Año Nuevo and 11% at Piedras Blancas (S1 Table in S1 File). We consistently searched both colonies for tagged adults during the winter breeding season, when females with pups hold their ground and allow observers closely. Tags were generally read with binoculars 3–5 m away from animals at Año Nuevo and with telescopes from bluffs 5–10 m away at Piedras Blancas. For this study we focus on pups tagged during 1994–2010. Because females start breeding at age 3–4 [16, 17], observations from 1997–2018 provide multiple opportunities to observe those cohorts during their breeding lifetimes. We assumed any female age 3 or older observed during the winter was breeding because 97.5% of adult females in the colony give birth [17, 18].

### Natal dispersal rate

Our focus is natal dispersal of females, defined as movement from the birth colony to a different colony on the first breeding attempt. The natal dispersal rate is the probability that a female alive for a first breeding attempt is at a colony different from her birth place. Any movements after initial breeding are termed adult dispersal, treated separately.

To estimate natal dispersal, define *T*_*i*_ as the total number of females tagged at colony *i*, and *B*_*i*_ the number of those observed breeding at any time in the future (S2 Table in S1 File lists mathematical symbols). Now consider *b*_*ij*_ as the subset of *B*_*i*_ first observed breeding at colony *j*. For breeding residents, ie not dispersing, *i* = *j*, while in dispersers *i* ≠ j. In this study, *i*, *j* ∈ (1, 2), and *B*_1_ = *b*_11_ + *b*_12_, *B*_2_ = *b*_21_ + *b*_22_. The ratio

$$\begin{array}{c} \mu_{ij}=\frac{b_{ij}}{B_{i}}=\frac{b_{ij}}{b_{ij}+b_{ii}} \end{array}$$ (1)

is a measure of natal dispersal rate from colony *i* to colony *j*. Any of the original *T*_*i*_ females never seen breeding do not enter into the calculation since we do not know whether or not they dispersed.

### Adult dispersal

Our goal is an analysis of natal dispersal, but we must address adult dispersal, defined as the movement between colonies after females begin breeding. If females move often as adults, estimates of natal dispersal will be confounded by adult dispersal in case females are not observed in their first breeding year. Preliminary calculations, though, showed adult dispersal to be rare [19], meaning that natal dispersal is effectively permanent dispersal: females spend entire lifetimes at a single colony. To confirm this, we present here an expanded estimate of adult dispersal based on observations of marked females across the 17 study cohorts. We found every case where a female was observed in consecutive breeding seasons and tallied the fraction of those that were at each of the two colonies. The proportion at a different colony in the second year is a direct estimate of annual adult dispersal.

### A detection bias

A concern with the calculation of dispersal is the failure to detect females that are present and breeding. Since our definition of natal dispersal is effectively lifetime emigration (given rare adult dispersal), it is lifetime detection that matters: the probability that a female who breeds one or more times during her lifetime is observed at least once. The concern here is that a difference in detection between our two study colonies will introduce a bias in dispersal estimates.

A simple example illustrates. Consider one cohort from colony 1 that ends up with 100 females breeding over their lifetimes, 90 resident where they were born and 10 dispersing permanently to a second colony, and a parallel cohort at colony 2 with 90 breeding as residents and 10 dispersing to colony 1. Both colonies have 10% dispersal. What if only half the females are detected over a lifetime at colony 1 but 80% at colony 2? Then the observed number of breeders born at site 2 and resident at site 2 would be *b*_22_ = 90 × 0.8 = 72, and the observed number emigrating would *b*_21_ = 10 × 0.5 = 5, leading to an estimate of dispersal from colony 2 to 1 of *μ*_21_ = *b*_21_/(*b*_21_ + *b*_22_) = 5/(5 + 72) = 0.065. The opposite calculation leads to *μ*_12_ = *b*_12_/(*b*_12_ + *b*_11_) = 8/(8 + 45) = 0.15. Dispersal toward the colony with fewer observations will be underestimated, and vice versa.

We can correct for this bias if we know lifetime detection probability at each study site. At Año Nuevo, we know that females are not always detected, and annual detection—the probability that a tagged female alive during one winter season is observed—was *δ* = 0.6 in a mark recapture analysis [20]. Here we show that *δ* can be estimated from observations of marked females, thus providing separate values for our two colonies. Then we show how to propagate from annual to lifetime detection probability, defined as Δ. Correcting for any difference in Δ leads to an unbiased comparison of dispersal between the two colonies.

### Estimating detection probability

Sightings of tagged females can be used to estimate annual and thus lifetime detection probabilities. First define rates of annual survival, *σ*, and tag retention, *ρ*. (Here we suppress subscripts, though every parameter is colony-specific; S2 Table in S1 File). In our definition, death includes permanent emigration outside the study colonies and tag loss means all tags are lost. There are previous estimates of survival and tag loss [18, 20], however, we demonstrate that they are not needed separately. Survival and retention appear in the calculations only as a product *τ* = *δρ*. Because it is the annual rate at which tagged females are back and observable after a year, we call it the return rate. Next, define a rate of reappearance, *π* = *δτ*, as the probability that a female returns with her tags and is detected. Thus

$$\begin{array}{c} \delta =\frac{\pi }{\tau }. \end{array}$$ (2)

Both *τ* and *π* can be derived directly from observations. S1 and S2 Appendices in S1 File give detailed derivations, but an intuitive grasp is straightforward. Return *τ* is the effective survival *σρ* (present and tagged), so it is the ratio of the number of animals alive at age *a* + 1 relative to a year earlier at age *a*:

$$\begin{array}{cc} \tau & =\frac{N_{a+1}}{N_{a}}=\frac{\delta N_{a+1}}{\delta N_{a}}. \end{array}$$ (3)

We cannot know either *N* directly, but we know *δN* because it is the number observed. Given the assumption that detection is constant from year to year and at all adult ages, we thus know the ratio *τ*. We overcome year-to-year variation in sighting effort by combining all ages across all years (S2 Appendix in S1 File).

The second required term, reappearance *π*, is the probability that an individual observed in one year is seen again the next year, because that requires survival, tag retention, and detection:

$$\begin{array}{cc} \pi & =\frac{D_{t+1}}{D_{t}}, \end{array}$$ (4)

where *D*_*t*_ is the number of all animals observed in year *t* and *D*_*t*+1_ the subset of those also seen in year *t* + 1. The distinction between these two equations is that the numerator in Eq (4) includes animals seen in both years while in Eq (3) it includes all seen in the second year, even those not seen in the first. Thus detection emerges from the ratio.

Eq (2) produces annual detection, but we need lifetime detection Δ. Finding Δ requires compounding multiple years of annual *δ*, ie the probability of failing to detect after two years is (1 − *δ*)^2^ etc. The full calculation (S3 Appendix in S1 File) leads to the following relation:

$$\begin{array}{c} \Delta =\frac{\delta }{1-\tau +\delta \tau }=\frac{\delta }{1-\tau (1-\delta )}=\frac{\pi }{\tau (1-\tau +\pi )}, \end{array}$$ (5)

giving Δ as a function of quantities known separately at each colony. The middle of the three forms is intended to offer some intuitive understanding, because the term 1/[1 − *τ*(1 − *δ*)] is the expected time until detection, showing that lifetime detection is annual detection multiplied by expected detection age.

### Correcting for lifetime detection

The number of tagged females observed breeding at least once over a lifetime can then be corrected for detection with

$$\begin{array}{c} {\hat{b}}_{ij}=\frac{b_{ij}}{\Delta_{j}}. \end{array}$$ (6)

Since the subscript *j* identifies the colony where these animals were observed, the formula includes Δ_*j*_, lifetime detection at *j*. The corrected $\hat{b}$ leads to a correction for dispersal rate

$$\begin{array}{c} {\hat{\mu }}_{ij}=\frac{{\hat{b}}_{ij}}{{\hat{b}}_{ij}+{\hat{b}}_{ii}}=\frac{b_{ij}/\Delta_{j}}{b_{ij}/\Delta_{j}+b_{ii}/\Delta_{i}}=\frac{b_{ij}}{b_{ij}+b_{ii}\Delta_{ji}}, \end{array}$$ (7)

using Eq (1) and defining Δ_*ji*_ = Δ_*j*_/Δ_*i*_ as the ratio of the detection probabilities.

This shows that the correction term for dispersal is based solely on the ratio of the two detection probabilities, and that dispersal in the opposite direction depends on the inverse Δ_*ij*_ = 1/Δ_*ji*_. Eq (7) is a quantitative statement of the qualitative conclusion that dispersal toward the well-observed colony is overestimated.

### Modeling and error propagation

A single estimate of the corrected annual dispersal is straightforward given Eqs 3, 4, 5 and 7. But a thorough estimate of error requires propagating through all intermediate calculations. A Bayesian approach allows this.

First, there is error in the number of observed females *b*_*ij*_. We assume these are binomial draws from the total number breeding, *B*_*i*_ = *b*_*ii*_ + *b*_*ij*_, so that *b* is a Poisson random variable

$$\begin{array}{c} b_{ij}∼\text{Pois}\left({\hat{b}}_{ij}\Delta_{ji}\right) \end{array}$$ (8)

and thus has known error. The term in parentheses is the model’s prediction for the number observed given dispersal rate and detection (Eq (6)). In the Bayesian framework, Eq (8) is a likelihood function for observations (*b*) given a hypothesis (${\hat{b}}_{ij}\Delta_{ji}$, ie the model).

But there is also error in lifetime detection, and it is based on several intermediate calculations of the rates *π* and *τ* (Eq (5)). The first depends on a ratio of integer counts, so the sampling distribution is binomial and a posterior distribution of the ratio *D*_*t*+1_/*D*_*t*_ is beta,

$$\begin{array}{c} \pi ∼\text{Beta}\left(D_{t+1},D_{t}\right). \end{array}$$

(The posterior is the probability of a given value of *π* given the data, the inverse of the likelihood function.) We created the posterior using the R function *rbeta* [21]. The second rate, *τ*, was derived from the slope of a regression from the age distribution (S1 Appendix and S1 Fig in S1 File). Standard linear regression leads to a slope *s* and its error *σ*_*s*_, thus

$$\begin{array}{c} \tau ∼\text{Norm}\left(s,\sigma_{s}\right) \end{array}$$

is a posterior probability of the desired parameter. We used the R function *rnorm* to generate it.

Here is where the value of the Bayesian approach arises. Random draws from the posteriors of *τ*, *π* were plugged into Eq (5) to produce a posterior distribution of lifetime detection at each colony, and those in turn were used to generate a posterior distribution of the ratio Δ_*ji*_. This final posterior became a prior probability in the model for dispersal (Eq (7)).

### A hierarchical model across years

A further feature we included with the Bayesian approach was to estimate a hyper-distribution across the annual dispersal parameters ${\hat{\mu }}_{ij}$. This is a valuable tool here because individual years did not have large samples, and the hierarchy allows annual estimates to differ while still supporting each other [22]. We assumed the values ${\hat{\mu }}_{ij}$ across years had a normal distribution described by hyper-parameters mean (*θ*_*ij*_) and standard deviation (Σ_*ij*_),

$$\begin{array}{c} {\hat{\mu }}_{ij}∼\text{Norm}\left(\theta_{ij},\Sigma_{ij}\right). \end{array}$$ (9)

There are separate hyperparameters *θ*_*ji*_, Σ_*ji*_ for dispersal in the opposite direction. We also tested a second multilevel model in which the mean *θ*_*ij*_ changed linearly through time, so *θ*_*ij*_ = *ν*_*ij*_ ⋅ *t* + *η*_*ij*_, where *t* is calendar year minus 2002. It has three hyper-parameters, slope *ν*_*ij*_, intercept *η*_*ij*_, and again Σ_*ij*_ for the residual standard deviation. In the absence of a significant effect of year on dispersal (ie *ν*_*ij*_ not different from zero), we would prefer the simpler model with only *θ*_*ij*_, Σ_*ij*_.

### Parameter estimation

The goal of the model is to generate estimates of dispersal parameters ${\hat{\mu }}_{ij}$ for 17 cohorts, plus hyper-parameters *θ*_*ij*_, Σ_*ij*_ (or *ν*_*ij*_, *η*_*ij*_, Σ_*ij*_ for the regression version). Models for dispersal in two directions were separate, so two models each have 19 (20) parameters. Because there are several steps in calculations, and given the hierarchical aspect, generating posterior distributions for these parameters is not as easy as inverting a likelihood function. It required a Markov Chain Monte Carlo sampling method (MCMC) based on the Metropolis algorithm [23]. This meant repeated draws of all parameters, with the likelihood recalculated each time. The Metropolis algorithm is a tool for keeping the MCMC parameter draws in the vicinity of the maximum, producing precisely a posterior distribution for every parameter. MCMC is a standard Bayesian method [20, 24, 25].

Sampling the parameters requires an inverted version of Eq (7),

$$\begin{array}{c} b_{ij}=\frac{B_{i}{\hat{\mu }}_{ij}\Delta_{ji}}{1-{\hat{\mu }}_{ij}\left(1-\Delta_{ji}\right)}. \end{array}$$ (10)

This gives the model prediction for the observed number of dispersers *b*_*ij*_ given the modeled ${\hat{\mu }}_{ij}$ and Δ_*ji*_; *B*_*i*_ is known. Define **Θ** as the full vector of 19 (or 20) parameters per colony. The MCMC chain was started with a full set **Θ** using observed ratios *μ* for $\hat{\mu }$. At every step, each of the 17 annual ${\hat{\mu }}_{ij}$ was plugged into Eq (10) (one-at-a-time) to predict 17 different *b*_*ij*_, the likelihood of each one found with Eq (8), then the likelihood of the hyper-parameters (given all 17 ${\hat{\mu }}_{ij}$) calculated from Eq (9). Combining all likelihoods was accomplished by summing their logarithms. Each step of the sampler involved a new selection of all parameters **Θ** at random, one at a time, recalculating all likelihoods, then using Metropolis [23] to decide whether to adopt the new values or keep the previous.

Embedded within the sampler was the prior distribution for lifetime detection, Δ_*ji*_, already completed and stored. At each step of the MCMC, one value from this prior was drawn at random. Thus Δ_*ji*_ was updated along with the other parameters **Θ**, but did not depend on the observations *b*, only the prior. No prior probability was used on any of the other parameters **Θ**, except for the trivial restrictions that probabilities $\hat{\mu }\in \left(0,1\right)$ and standard deviations Σ > 0.

We executed samplers for 6000 steps and discarded the first 2000 as burn-in. Parameter chains converged quickly and consistently. The mean of post-burn-in chains was used as the best estimate for every parameter and the central 95^th^ percentiles as credible intervals.

### Inference on dispersal rate and colony size

The Piedras Blancas colony grew by 15-fold over the course of the study (S1 Table in S1 File). In early years, breeding females would have found relatively unoccupied beaches, in contrast to later years, when arriving animals found many beaches occupied. We do not know, however, whether colony density (females per beach area) increased with colony size, because animals spread out as the colony expanded. In contrast, the Año Nuevo colony was relatively stable, declining by 22% over the same period (S1 Table in S1 File), and seals occupied the same beaches throughout. We tested two hypotheses about the importance of colony size. First, we asked whether dispersal from Piedras Blancas increased through time using the hyper-parameter *ν*, the slope of the regression between dispersal and year. If its credible intervals overlapped zero, we would reject the influence of colony size. Second, we compared dispersal from Año Nuevo toward Piedras Blancas and vice versa using credible intervals to test the prediction that dispersal was more frequent from the large colony to the small. The hyper-means *θ* offered a test of the overall rates, plus there was a test every year based on credible intervals of the annual $\hat{\mu }$.

## Results

### Observed natal dispersal

Observed dispersal was more frequent from Piedras Blancas to Año Nuevo than vice versa (Table 1). Combining all 17 cohorts, there were 468 pups tagged at Piedras Blancas that were observed as breeding adults: 344 at their birth colony and 124 that emigrated to Año Nuevo (26.5% emigrated). There were 601 pups from Año Nuevo that were observed breeding: 566 as residents and 35 as emigrants to Piedras Blancas (5.8% emigrated). But this comparison is biased by detection, and the correction requires estimates of lifetime detection.

**Table 1:** Observed dispersal within annual cohorts of female elephant seals. Tagged: total number of females tagged as pups each year at the two colonies. Breeding: the number of those observed breeding at least once over their lifetimes. Resident: subset of breeders that were first seen breeding where they were born. Emigrant: subset of breeders first seen breeding at the opposite colony.

Birth year	Año Nuevo born				Piedras Blancas born			
	Tagged	Breeding	Resident	Emigrant	Tagged	Breeding	Resident	Emigrant
1994	240	24	24	0	120	13	11	2
1995	421	55	52	3	152	40	23	17
1996	271	51	46	5	148	41	33	8
1997	210	16	15	1	14	3	3	0
1998	211	37	36	1	155	35	23	12
1999	146	21	21	0	158	24	16	8
2000	264	46	45	1	156	32	14	18
2001	108	16	14	2	158	45	29	16
2002	138	40	39	1	158	42	36	6
2003	177	46	39	7	138	29	22	7
2004	168	25	23	2	150	31	31	0
2005	378	42	36	6	176	16	15	1
2006	286	28	28	0	132	20	15	5
2007	366	56	54	2	176	31	25	6
2008	214	26	25	1	174	22	18	4
2009	298	46	44	2	123	16	10	6
2010	208	26	25	1	196	28	20	8
Total	4104	601	566	35	2486	468	344	124

### Lifetime detection

At Piedras Blancas, the estimated lifetime detection was Δ = 0.549, while at Año Nuevo, Δ = 0.923, a highly significant difference (credible intervals in Table 2). The ratio of 0.598 (Table 2) means that the average female spending a lifetime breeding at Piedras Blancas was 60% as likely to be detected as a similar female at Año Nuevo. The observed reappearance rate, *π*, drove the difference: 16% of females seen in one year at Piedras Blancas were detected the next year, compared to 57% at Año Nuevo (Table 3). Since return rates *τ*—survival and tag retention—were indistinguishable between the colonies, the difference in annual detection matched the difference in reappearance (Table 2).

**Table 2:** Parameter estimates from the Bayesian dispersal model. The first five parameters are colony-specific, but detection ratio and mean dispersal relate the two colonies and include subscripts 12 to show this. Under the Año Nuevo column, detection ratio is Año Nuevo divided by Piedras Blancas, while dispersal is from Año Nuevo to Piedras Blancas. The Piedras Blancas column has the opposite. For each, the best estimate is followed by 95% credible intervals in parentheses.

Rate	Symbol	Colony	
		Año Nuevo	Piedras Blancas
Reappearance	*π*	0.563 (0.54,0.58)	0.165 (0.14,0.19)
Return	*τ*	0.799 (0.79,0.81)	0.783 (0.75,0.81)
Annual detection	*δ*	0.704 (0.68,0.73)	0.211 (0.18,0.25)
Lifetime detection	Δ	0.922 (0.91,0.93)	0.551 (0.49,0.61)
Year effect	*ν*	0.012 (-0.09,0.11)	-0.024 (-0.11,0.06)
Detection ratio	Δ_12_	1.678 (1.52,1.87)	0.598 (0.54,0.66)
Mean dispersal	*θ*_12_	0.0799 (0.05,0.11)	0.1590 (0.11,0.22)

**Table 3:** Reappearance rate of breeding females. The total sample is the sum of all lifetime breeding records of females in the study cohorts through year 2017 (ie excluding 2018); any female seen more than once counts every time. The two rows divide the total sample based upon breeding location (birth location is not relevant here). For example, the 2297 breeding records at Año Nuevo is the sum of all records of 601 females born at Año Nuevo and 344 females born at Piedras Blancas (numbers in Table 1). There are a few individual females counted in both rows because of adult dispersal. Location in year 2 subdivides each row based on where females were observed one year later, none meaning not seen anywhere; they add up to the total. The reappearance rate, *π*, is the fraction reappearing at the same colony.

Location in year 1	Total sample	Location in year 2		
		Año Nuevo	Piedras Blancas	None
Año Nuevo	2297	1293	8	996
Piedras Blancas	671	2	110	559

### Adult dispersal

Of more than 1400 observations of breeding attempts in consecutive years, only 10 (0.7%) included females moving to the opposite colony in the second year (Table 3). Two moved from Piedras Blancas to Año Nuevo and eight the opposite.

### Corrected natal dispersal

Higher lifetime detection probability at Año Nuevo means that the observed number dispersing toward Año Nuevo (Table 1) is biased upward. Yet even after the correction, dispersal northward from Piedras Blancas was double that southward from Año Nuevo, 0.16 versus 0.08 (Table 2). The 95% credible intervals just met (Table 2).

**Fig 2:**
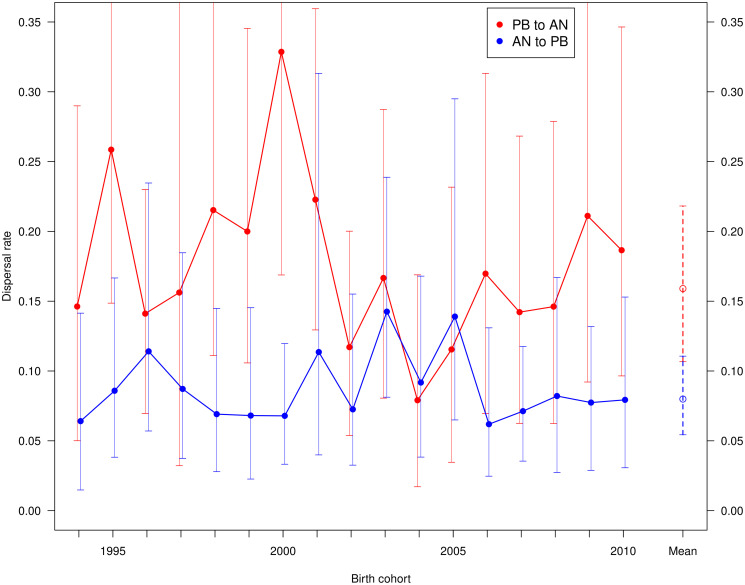
Natal dispersal by birth cohort. Dispersal rate of juveniles born at Piedras Blancas (PB) to breed at Año Nuevo (AN) in red. Dispersal from Año Nuevo to Piedras Blancas in blue. Best estimates are filled circles, and 95% credible intervals are vertical lines. The overall mean across all cohorts are the rightmost points with dashed credible intervals (Table 2).

There was considerable year-to-year variation in the rate from Piedras Blancas, indeed the rate of 0.41 for the cohort born in 2000 was significantly higher than the rate of 0.17 in 2004 (Fig 2). There was much less variation in the southward direction, and none was significant (Fig 2).

### Dispersal and density

There was no temporal trend in dispersal in either direction (parameter *ν* did not differ from zero, Table 2). During the early years of the study, when the Año Nuevo colony had far more animals than Piedras Blancas, dispersal was higher from Piedras Blancas toward Año Nuevo, and the northward rate in 2000 was significantly higher than the southward (Fig 2).

## Discussion

During the 1970s and 1980s, young elephant seals in California were observed emigrating from their birth colonies to establish new colonies [26, 27], but rates of dispersal were not estimated. Our results here confirm dispersal and add quantitative results over many years. Juvenile females dispersed between two major colonies 200 km apart at a substantial rate, 16% in the northward direction and 8% southward. Other published rates for pinnipeds are lower. In harbor seals (*Phoca vitulina*), 10% of breeding females emigrated to colonies within 10 km of their birthplace, but none dispersed more than 20 km [28], while in gray seals (*Halichoerus grypus*), the fraction dispersing was < 5% among all colony pairs, though that was based on population models, not direct observations [29]. Interestingly, a literature review covering many species (but no marine mammals), the mean fraction dispersing was 15% [10], close to what we observed. How dispersal was measured, and observed distances, were extremely variable among species, so this is not a safe generalization.

Dispersal rates between Año Nuevo and Piedras Blancas remained consistently high over 17 consecutive cohorts, and though there were fluctuations, there was no long-term trend. During that period, the population at Piedras Blancas grew 15-fold while the number at Año Nuevo declined slightly. There was thus no indication that emigration increased with colony size. Moreover, during the mid-1990s, there was more dispersal from the smaller Piedras Blancas colony toward the larger at Año Nuevo. These results do not support earlier observations that colony size drove decisions of young females to emigrate to found new colonies [7]. Those observations were based on the Año Nuevo Island colony, where females are tightly packed in one large group and most individuals encounter high density of near neighbors. In contrast, the Piedras Blancas colony spread to new beaches as it grew, so it is not clear whether individuals faced higher density after 2010 than in the 1990s. In a 2005 review of studies examining emigration and population density, Matthysen [12] found a positive relation in some studies but not others. A recent study of falcons (*Falco sparverius*) found no correlation between population density and frequency of dispersal [9].

If our study had ended with the cohort of 2005, we might have concluded that emigration from Piedras Blancas was declining after the high rates of 1995 and 2000, opposing the expectation that emigrants escape high density. Perhaps small size of the colony in the 1990s made it less attractive because elephant seals prefer dense aggregations. After 2005, however, emigration from Piedras Blancas rose again, and it is possible that positive and negative effects of density were both at work, or, more likely in our view, dispersal decisions are based on factors other than colony size.

There was directionality in dispersal, with the northward rate double the southward. The difference was at the margin of statistical significance, and it should be further tested, but the northward flow fits the elephant seal migration. Females move northwestward from their breeding colonies in California toward distant foraging grounds [30, 31], thus animals born at Piedras Blancas pass Año Nuevo on the migration, but not vice versa. The feeding grounds are considerably farther than the distance between colonies, and from that perspective dispersal distances in elephant seals are not high. A tendency for long-distance migrants to disperse well compared to non-migrants has been demonstrated in birds [32].

Our dispersal estimates include thorough analysis of error, most importantly the bias caused by unequal detection probabilities. This bias is often mentioned in studies of bird dispersal but is difficult to quantify [1]. We were able to estimate lifetime detection from many years of resightings of long-lived females, and we demonstrated much higher detectability at Año Nuevo than Piedras Blancas. Better detection at Año Nuevo can be attributed to better beach access as well as student participation in the university-sponsored research.

These natal dispersal rates may be underestimates because we only included two colonies, and we have evidence of movement to additional colonies. In Condit et al. [20], we reported the lifetime fate of three cohorts of females branded at Año Nuevo during the 1980s, including emigrants to two adjacent colonies. We found that seven emigrated of the 37 that bred (18.9%); this was before the Piedras Blancas colony existed, and the emigrants were at the Farallon and Point Reyes colonies north of Año Nuevo. In an analysis of three cohorts [19], all four colonies were included. No correction for detection was incorporated, but the observed number of emigrants from Piedras Blancas to the other two colonies was nearly as high as the number moving to Año Nuevo, while the number dispersing from Año Nuevo to the other two was even higher than the number moving to Piedras Blancas. Moreover, there may be dispersal to the Channel Island colonies, 200 km south of Piedras Blancas, but observations at those large island colonies are much more difficult. Overall, we have reason to believe that total natal dispersal rates are even higher than those reported here, perhaps reaching 20%.

Once breeding, adult females move at much lower rates. In the cohorts we studied, fewer than 1% moved over consecutive years. Northern elephant seal colonies are thus well-mixed genetically in that juveniles move among them at substantial rates, but most adults spend their lives at a single colony. Since all the colonies, including Mexico, form a chain with gaps of no more than 500 km, this mixing undoubtedly includes the entire range. In terms of response to the environment or prey abundance [33], we conclude that the northern elephant seal should be treated as one large population.

Perhaps most important is the role of dispersal in recovery from population bottlenecks, and this is clear in northern elephant seals. Nearly extinct in 1892 [4], the species refilled its range within 70–80 years [6, 34]. We demonstrate here that dispersal continues now, even after the range has filled, and elephant seals are thus poised to expand their range further, as they recently have at King Range in northern California [35]. In other pinnipeds, poor dispersal may be a primary reason for slow recovery [11]. The fact that elephant seals disperse well appears to be a fortuitous trait in the face of the 19^th^ century decimation by hunters.

## Supporting information

S1 File Three appendices provide complete derivations of the estimates of annual and lifetime detection of breeding females. Table S1 gives the number of animals tagged relative to those born at each colony, and Table S2 is a full list of mathematical symbols used. Figure S1 shows the rate of decay of the number of tagged females versus age at both colonies, used in estimating return rate *τ*.
(PDF)
