# Supplementary material for: Quantifying dispersal between two colonies of northern elephant seals across 17 birth cohorts
Source: PLoS One. 2023 Nov 30;18(11):e0288921. doi: 10.1371/journal.pone.0288921 (PMC10688689; doi:10.1371/journal.pone.0288921)

Quantifying dispersal between two colonies of northern elephant seals across 17 birth cohorts: Supplement

Richard Condit[1*], Brian Hatfield[2], Patricia A. Morris[3], Daniel P. Costa[3],

1 Department of Ecology and Evolutionary Biology, University of California, Santa Cruz, USA

2 USGS Western Ecological Research Center, 100 Shaffer Road, Santa Cruz, CA

3 Institute for Marine Sciences, University of California, Santa Cruz, USA

* condit@gmail.com

Appendix S1: Rate of return

The first step in computing lifetime detection probabilities at the two colonies is an estimate of what we define as the rate of return of breeding females from year to year. This is the probability that a female alive in year t is still alive, has not emigrated, and has a tag in year $t+1$, so return rate $\tau = \sigma\rho$ (Table S2). Under reasonable assumptions, τ can be estimated from observations of tagged animals. The annual detection term δ is not needed in these calculations given the assumption that δ is constant from year-to-year and throughout the breeding lifetime, until senescence. Lifetime survival curves suggest that both σ and δ are constant, with senescence setting in at age 17 [**?**]. Female behavior on the breeding colony is consistent at all ages 5 and above, also supporting a constant detection term. We do not have independent information on how tag retention, ρ, changes with age; we return to this later.

Start with a population of N_0 females breeding for the first time. Then $D_0 = \delta N_0$ is the number of those observed. The subscript refers to an age, defining age=0 as the first year each female breeds. For animals one year older, the number returning (alive and tagged) is $N_1 = \sigma\rho N_0 = \tau N_0$. The number of those detected is $D_1 = \delta N_1 = \delta\tau N_0$. At subsequent ages,

$$\begin{aligned} D_a &= \delta N_a \\ &= \delta\tau N_{a-1} \\ &= \delta\tau^{a-1} N_0. \end{aligned} \tag{S1}$$

The exponent is $a-1$ because we define the initial cohort as females alive and breeding for the first time at $a=0$, so the survival probability until year a requires $a-1$ survival steps. Taking logarithms,

$$\ln D_a = [\ln\delta + \ln N_0 - \ln\tau] + [\ln\tau]\cdot a. \tag{S2}$$

The two terms in square braces are constants that can be estimated from a regression of $\ln D_a$ against a. That is, the number of animals observed at successively greater ages declines exponentially with time, with slope $\ln\tau$ (Fig. S1). We thus have a simple way

of estimating τ. Detection δ appears in the intercept but not the slope based on the assumption that δ is constant during females' breeding years, so its impact cancels out.

Given the main breeding years described above, we calculated the age distribution from ages 5-15 to estimate this regression (Fig. S1). The regression slopes at the two colonies barely differed and were statistically indistinguishable (Table 2, main text). In both, slopes were slightly steeper after age 10, but not significantly so (Fig. S1). In Condit et al. [?], we demonstrated constant adult survival until age 17, and though we have not estimated tag loss in older animals, it evidently does not change much.

Appendix S2: Annual detection

The next requirement for finding lifetime detection of breeding females is an estimate of annual detection probability, δ. This can be derived from observations of tagged females in consecutive years, given the estimate of return rate τ (Appendix S1). Here, define N_0 as the number of tagged adult females breeding in year 0 (in contrast to Appendix S1, where the subscript referred to age, not year). We observe $D_0 = \delta N_0$ of those animals. A year later, the number of those D_0 returning is τD_0, and using the assumption that detection probability is equal in the two years, the number of those detected is $D_1 = \delta\tau D_0 = \pi D_0$. The fraction $\pi = D_1/D_0$ is the reappearance rate as defined in the main text (Table S2). Then

$$\delta = \frac{\pi}{\tau} = \frac{D_1}{D_0\tau}. \tag{S3}$$

Notice that D_1 is not all animals detected in year 1, it is only those detected in year 1 that had also been detected in year 0. It does not include animals observed in year 1 but not observed in year 0. In contrast, the successive ratios of Equation S2 and Figure S1 are based on all animals seen each year.

Appendix S3: Lifetime detection

Now we derive the probability that a breeding female elephant seal is detected at least once in her lifetime, given that she bred at least once. Again, we use the annual return rate, τ, which is the product of annual survival σ and tag retention ρ, and annual

detection δ. We rely on the assumption that all three are constant through the adult lifetime, until the age of senescence. We also must assume that a female's detection in one year is independent of her detection in other years; we do not have evidence to support this assertion.

Female elephant seals can breed many years, and assuming independence from year to year, long-lived females will almost certainly be detected. Lifetime non-detection is most likely in females that breed once then die. The full derivation requires the probability of breeding once then dying, breeding twice then dying, etc.

It is easier to work with non-detection probabilities, so define annual non-detection as $\lambda = 1 - \delta$ and lifetime non-detection as $\Lambda = 1 - \Delta$. The calculation begins with a group of females that are present on the breeding colony for the first time in their lives, N_0, exactly as in Appendix S1. Consider the subset of this group that returns in exactly n breeding seasons, then dies (or emigrates or loses tags), so there are n chances to detect this group. Failure to detect in every one is λ^n, so the probability of lifetime non-detection over exactly n years is

$$\Lambda(n) = \lambda^n. \tag{S4}$$

Since females have variable lifetimes, we need to calculate the probability of each lifetime n. Call $P(n)$ the probability that the average female returns in exactly n breeding seasons, disappearing by $n + 1$. Because we assume the initial cohort was already alive in the first year, $n - 1$ return events are required. The probability is thus

$$P(n) = \tau^{n-1}(1 - \tau). \tag{S5}$$

τ is the annual return rate (Appendix S1, Table S2).

We need this calculation for every lifespan: those females returning exactly $n = 1, 2, 3, ...$ years. Each of those groups includes a proportion of females given by S5, and each is subdivided into two smaller groups, those not detected and those detected, the former from (Eq. S4). The product of Equations S4 and S5 is the proportion of females with a given lifetime that were never detected, so we sum those products over all lifetimes

to find the total proportion never detected,

$$\Lambda = \sum \Lambda(n)P(n) = \sum_t \lambda^t \tau^{t-1}(1-\tau)$$
$$= \lambda(1-\tau)\sum_t (\tau\lambda)^{t-1}. \tag{S6}$$

The summation runs from $t = 1$ to $t = \infty$ (years). The final version is rearranged so that the two terms inside the summation have the same exponent, $t - 1$. Then Equation S6 is an infinite geometric series, each term a factor $\tau\lambda$ times the previous. In reality, the series would have to be curtailed when senescence starts, but the size of the seventeenth term is vanishingly small, so the infinite approximation is very close. Since we need lifetime detection, $\Delta = 1 - \Lambda$, the formula for a geometric series [[**?**]] yields

$$\Delta = 1 - \frac{\lambda(1-\tau)}{1-\tau\lambda}$$
$$= \frac{1-\lambda}{1-\tau\lambda}$$
$$= \frac{\delta}{1-\tau+\delta\tau} \tag{S7}$$
$$= \frac{\pi/\tau}{1-\tau+\pi}. \tag{S8}$$

These give the lifetime detection probability as a function of the annual detection, δ, annual return, τ, and annual reappearance, π.

Table S1. Number of females weaned and tagged each year of the study at both colonies, Año Nuevo (AN) and Piedras Blancas (PPB). Number weaned is from published tables [?, ?], divided by two (assuming females are half those weaned). The number tagged means females; it includes half of a small number whose sex was not recorded (Table 2, main text).

	Weaned		Tagged		Fraction tagged	
Year	PPB	AN	PPB	AN	PPB	AN
1994	146	1024	120	240	0.822	0.234
1995	302	1116	152	421	0.503	0.377
1996	494	1123	148	271	0.300	0.241
1997	598	1160	14	210	0.023	0.181
1998	836	1128	155	211	0.185	0.187
1999	956	1100	158	146	0.165	0.133
2000	923	1108	156	264	0.169	0.238
2001	970	1060	158	108	0.163	0.102
2002	1088	1124	158	138	0.145	0.123
2003	1324	1202	138	177	0.104	0.147
2004	1526	1010	150	168	0.098	0.166
2005	1757	1222	176	378	0.100	0.309
2006	1894	1201	132	286	0.070	0.238
2007	2040	1161	176	366	0.086	0.315
2008	2004	1072	174	214	0.087	0.200
2009	1904	1006	123	298	0.065	0.296
2010	2234	922	196	208	0.088	0.226

Table S2. Math symbols used. Latin letters are observed or predicted counts of animals; Greek letters are parameters to be estimated (σ and ρ are only estimated as a product). Subscript i refers to colony; parameters with i alone are colony-specific. Those with ij relate two colonies, i and j.

Symbol	Definition
T_i	Number of animals tagged as pups
B_i	Number of T_i later observed breeding (at any colony)
b_{ij}	Number of animals born at i then first observed at j ($i = j$ or $i \neq j$)
D_{ai}	Number of animals detected breeding, age (or year) a
N_{ai}	Number of animals alive, age (or year) a
σ_i	Annual survival rate
ρ_i	Annual tag retention rate
δ_i	Annual detection probability
τ_i	Annual return rate ($= \sigma_i \rho_i$)
π_i	Annual reappearance rate ($= \delta_i \sigma_i \rho_i$)
Δ_i	Lifetime detection probability
Δ_{ij}	Lifetime detection ratio, colony i to j
λ_i	Annual non-detection probability (=1-δ_i)
Λ_i	Lifetime non-detection probability (=1-Δ_i)
μ_{ij}	Uncorrected dispersal rate from i to j ($= b_{ij}/[b_{ij} + b_{ii}]$)
$\widehat{b}_{ij}$	Number of animals expected given lifetime detection ($= b_{ij}/\Delta_j$)
$\widehat{\mu}_{ij}$	Annual dispersal rate corrected for lifetime detection ($= \widehat{b}_{ij}/[\widehat{b}_{ij} + \widehat{b}_{ii}]$)
θ_i	Hyper-mean of corrected dispersal (overall mean across years)
Σ_i	Hyper-standard-deviation of corrected dispersal
ν_i	Slope from regression between annual dispersal rate and year
η_i	Intercept from same regression
Θ_i	Vector of all parameters in model (annual dispersal, hyper-parameters)

Figure S1. Decline in number of observed females on a colony with age. The vertical axis gives the natural log of the number of animals observed at each age, combining cohorts 1994-2010. The horizontal axis is female age in years; ages 5-15 include the main breeding life, when fecundity is at its maximum and before senescence. A) Año Nuevo. B) Piedras Blancas. The slopes provide estimates of $\ln\tau$, the logarithm of the rate of return (Appendix S1); $\tau = 0.799$ at Año Nuevo (standard error 0.0056), $\tau = 0.783$ (standard error 0.015) at Piedras Blancas. At each colony, a separate slope was fitted using ages 5-10 and again ages 10-15 (dashed gray lines). In both cases, the second slope was steeper, but in neither case did it differ statistically from the first.

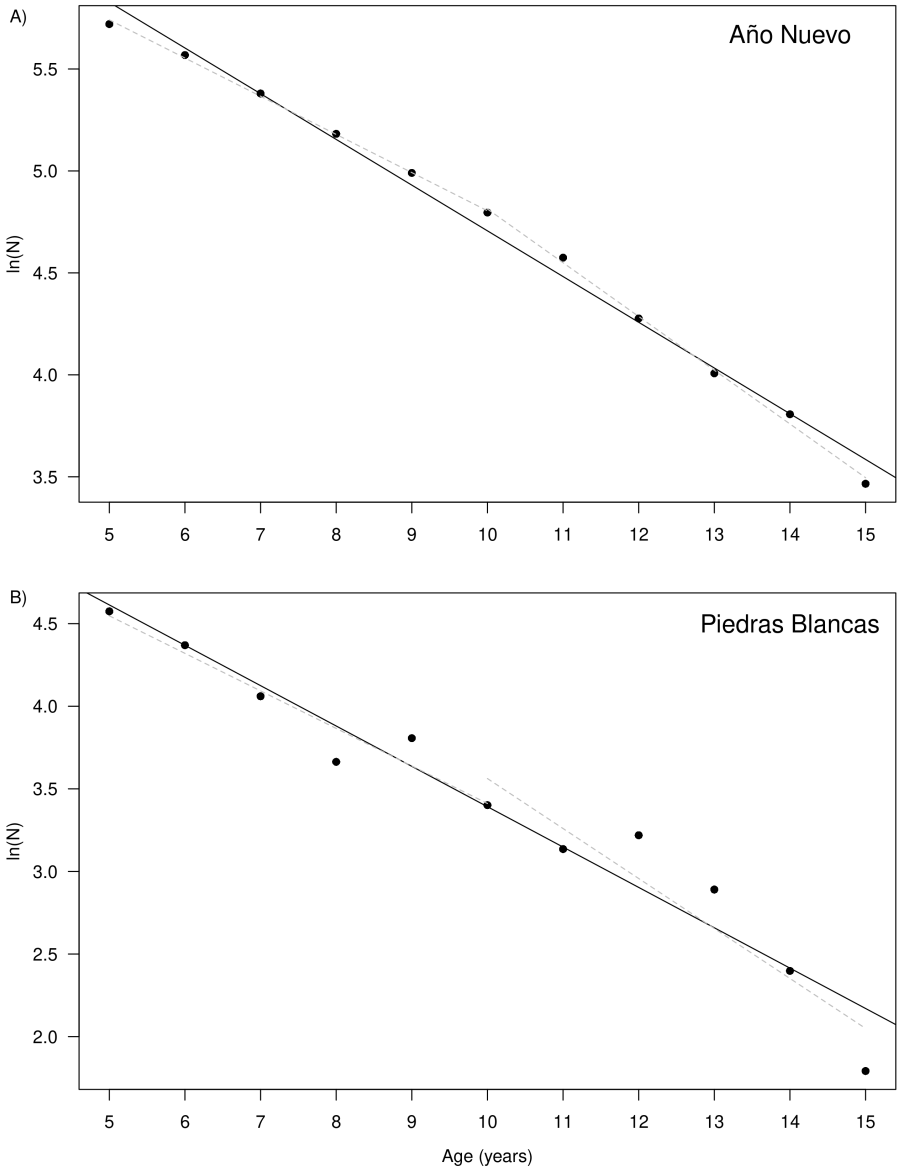

Supplement: S1 File — Table S1 gives the number of animals tagged relative to those born at each colony, and Table S2 is a full list of mathematical symbols used. Figure S1 shows the rate of decay of the number of tagged females versus age at both colonies, used in estimating return rate τ. (PDF) [file pone.0288921.s001.pdf]
